# Modern Therapeutics of Diseases of Children

**Published:** 1885-07

**Authors:** 


### Book Reviews.

---

Modern Therapeutics of the Diseases of Children, with Observations on the Hygeine of Infancy. *By* Joseph F. Edwards, m. d., etc., etc. *Philadelphia:* D. G. Brinton. *8vo. 346 pp.*

This excellent compendium of the therapeusis of childhood is issued by the same publisher and with a similar arrangement to that of his "Therapuetics of Gynecology and Obstetrics," which is that of an alphabetical list of diseases with the various treatment suggested for each. In addition to this, there is a check-list of drugs under each head, also alphabetically arranged and starred according to Waring's original plan. Whether these stars have always been affixed to the most reliable remedies for the disease treated, often admits of discussion, as in the case of quinine for true croup and carbolic acid in pertussis; but surely he must be a captious practitioner who cannot find something appropriate in the generous list of drugs suggested. As is always the case, the more uncertain the disease, the more bewildering the number o remedies proposed. For instance, under the head of diphtheria we find more than fifty remedies and the wildest contradictions in regard to treatment. Drysdale informs the reader that chlorate of potash is the most valuable of all remedies in diphtheritis, if given without timidity, while Herroch and others, especially warn us against large doses of the same. Calomel, he says, on page 150, is almost discarded in treatment, and on pages 153, 165 and 180, he recommends strongly its use, in one case in large doses. *Quot medici, tot sententiæ* regarding diphtheria, even to German nihilism, which asserts all remedies except sulphur "are entirely useless in severe cases, and these alone should be condemned since the mild cases recover spontaneously," (p. 153). In a book of this character such a statement ought, at least, to be offset by the teachings of Professors Pepper and Jacobi on this subject, but we look in vain for them. In fact, the most serious objections that can be brought against the work is the relatively small importance given some of our best American authorities in pædiatrics. Why, for instance, should Herroch, of Berlin, be quoted forty times and Drs. Jacobi, Plant and Holt given only a single reference, and Armand Semple, of London, thrice the prominence of J. Lewis Smith, of New York? It certainly awakens a suspicion that the preface, to the contrary notwithstanding, the book has been scissored from only a limited number of authorities and a restricted file of medical journals. Nevertheless, the book has many new and valuable points for one interested in the therapeutics of childhood. The subject of the hygeine and general treatment of infancy contains much that can be found elsewhere in no other single work, though the addition of Young's posology and Ellis' division of drugs would have enhanced its value. The list of diseases contains several that are rarely met with in this country, but unfortunately, omits all treatment of such common troubles of children as adenitis, œsophagitis, ring-worm, constipation and all affections of the eyes, ears, or heart.

The type and paper of the book are good, and it is remarkably free from the typographical errors so usual in such collections of prescriptions. It is also provided with good indices of diseases, remedies and authors referred to. Among the latter we note four of our Chicago physicians, and from internal evidence furnished by the prescription on page 292, conclude that the Dr. Davis, of New York, there quoted really resides in Chicago. M. P. H.

